# Patient Screenings for Preconception Health Interventions at a Community Pharmacy

**DOI:** 10.3390/pharmacy8040181

**Published:** 2020-10-05

**Authors:** Alex J Luli, Natalie Tran, Angela Ataya, Sally Rafie

**Affiliations:** 1 UC San Diego Skaggs School of Pharmacy and Pharmaceutical Sciences, La Jolla, CA 92093, USA; n9tran@ucsd.edu (N.T.); aataya@ucsd.edu (A.A.); srafie@ucsd.edu (S.R.); 2 The Pharmacists Clinic, San Diego, CA 92122, USA

**Keywords:** preconception health, clinical pharmacist, community pharmacy, contraception

## Abstract

Preconception health refers to health before pregnancy and involves addressing risk factors that can negatively impact either a patient or future pregnancy. Pharmacists can play an essential role in screening for and managing patients’ risk factors to optimize pregnancy outcomes. The primary objective of this study is to determine whether preconception health screenings in community pharmacy settings identify opportunities for preconception health services, particularly pharmacy-based interventions. The secondary objectives are to describe the preconception health status of community pharmacy patients and their interest in receiving preconception care services by a clinical pharmacist in a community pharmacy setting. Two independent pharmacies conducted a pilot project where people were invited to complete a health screening form that evaluated their preconception health. Participants received a personalized health report with an invitation to meet with the clinical pharmacist for services related to identified opportunities, such as contraception and immunizations. Retrospective analysis was conducted for data collected from 43 women during the patient screening effort in three community pharmacy settings (two independent community pharmacy locations and one neighborhood pharmacy outreach event). Nearly all participants (*n* = 42, 98%) had at least one opportunity identified to receive preconception care services, with the majority related to their alcohol use (60%). A majority of participants (56%) indicated an interest in learning more about preconception services offered at the pharmacy, but only 19% wanted to schedule an appointment with a pharmacist. Thus, there is an apparent need and opportunity for utilization of preconception health services at the pharmacy.

## 1. Introduction

Preconception care can be defined as individualized care for people that is focused on reducing maternal and fetal morbidity and mortality, increasing the chances of conception when pregnancy is desired, and providing contraceptive counseling to help prevent unintended pregnancies [1]. While planning for a pregnancy, the United States Centers for Disease Control and Prevention (CDC) recommends talking to a health care provider about medical conditions, lifestyle, behaviors, medications, and vaccinations—before becoming pregnant [2]. In addition, the American College of Obstetricians and Gynecologists (ACOG) and the American Society for Reproductive Medicine (ASRM) also recommend that any patient encounter with nonpregnant women or men with reproductive potential is an opportunity to counsel about wellness and healthy habits, which may improve reproductive and obstetric outcomes should they choose to reproduce [3]. This is especially important since nearly half (45%) of all pregnancies in the United States were unintended in 2011 [4]. Further, most birth defects occur within the first three months of pregnancy, and nearly one-fifth of pregnant women in the United States receive no prenatal care before the end of their first trimester [5]. Lack of adequate preconception care may be further compounded in certain subpopulations, where additional disparities in unintended pregnancy rates exist, such as individuals who are between ages 18 and 24, financially unstable, or cohabiting with rates two to three times that of the national rate [4]. 

Maternal factors including, but not limited to, tobacco use, lack of folic acid supplementation and diabetes have been associated with infants with low birthweight [6], neural tube defects [7,8], and fetal death [9], respectively. Further, the CDC recommends that women 15–45 years of age get 400 mcg of folic acid every day to prevent neural tube defects [10]. One study found almost half of U.S. women reported enriched cereal grain products as their only source of folic acid, and up to 700 additional neural tube defects could be prevented per year with additional folic acid intake [8]. Hence, it is essential to advocate for healthy behaviors and improve overall well-being for people of reproductive potential to increase the chances for healthy pregnancies. While awareness of preconception health is low, many women are willing to adopt healthier behaviors during the preconception period if recommended by a healthcare professional [11]. Provider encouragement and active offers of preconception care has been shown to facilitate its uptake [12]. Despite this, one study found that women are not readily being offered preconception health services by their health care providers. Out of 499 women surveyed, a vast majority (95.3%) preferred to receive preconception health information from their primary care provider, but only 39% could recall their physician ever discussing it with them [13]. As such, preconception care may be increased with more encouragement and active offers by providers. Pharmacists are well positioned in the community to promote and provide consultation on preconception health. 

As one of the most accessible healthcare providers, community pharmacists can play a vital role in providing preconception care services to the general public. Almost 90% of Americans live within five miles of a community pharmacy [14]. In addition, many pharmacies are open evenings, weekends, and holidays. This accessibility has helped pharmacists to make positive impacts on public health. For example, expansion of pharmacist-provided immunizations has given Americans a trusted and convenient option to receive vaccinations [15,16,17]. In another example, allowing pharmacists to dispense naloxone, an opioid-reversal agent, without a prescription in many states has increased access to a potentially life-saving medication. In Ohio, the implementation of this law was associated with a significant increase (2328%) in naloxone dispensing rates [18]. Similarly, within the current pharmacist scope of practice, there are increasing opportunities for pharmacists to improve preconception care [19,20,21,22,23,24,25,26]. These roles include providing education, counseling, and/or services to address family planning. In addition, pharmacists are well positioned to help with medication and disease state management, immunizations, health screenings, and substance use [19]. Further, there have been emerging roles for pharmacists in the direct provision of hormonal contraception, including emergency contraception, to women without a prescription [27,28,29,30]. As of 2020, there are 13 U.S. jurisdictions, including the District of Columbia, that have authorized pharmacists to specifically prescribe hormonal contraception under statewide protocol or standing order without a prior prescription [31]. A national survey of pharmacists and pharmacy students found that most respondents are either currently providing or interested in providing some form of preconception care services [32]. Moreover, a survey looking at pharmacists’ attitudes regarding reproductive health services indicated a majority (57%) believe that oral contraceptive methods should be pharmacist-prescribed [33]. Hence, aside from a demonstrated need for preconception care, pharmacists are interested in providing such services. 

A previous study evaluated whether a preconception health screening tool in a community pharmacy could assess preconception health service opportunities [34]. This study aims to further determine if these findings are reproducible in a different geographical setting, expand the patient risk factors assessed, and understand barriers to the utilization of preconception care services in a community pharmacy setting. 

The primary objective of this study is to determine if screening people of reproductive age in community pharmacy settings identifies opportunities for preconception care services, defined by lack of contraception use, or the presence of one or more risk factors identified in the CDC report on Improving Preconception Health and Health Care [35]. The secondary objectives are to describe participant preconception health status and their interest in receiving preconception care services by a clinical pharmacist in a community pharmacy setting. 

## 2. Materials and Methods

This study is a retrospective analysis of data collected during a pilot patient screening effort at two independent community pharmacies under the same ownership in San Diego, CA and one pharmacy outreach event between September 2017 and March 2018. These pharmacy locations were selected based on their preconception health services offered by community pharmacists including but not limited to: contraception, immunizations, supplements, tobacco cessation, medication administration and consultation. The pharmacy participated in a local, community outreach event that was open to the public and designed to raise awareness about alcohol-free pregnancies. Potential participants were asked to complete a preconception screening questionnaire entitled “Young Women’s Health Screening Form” (see Supplemental Material) at one of the two pharmacy locations during routine pharmacy activities, or during one outreach event in the neighborhood if they met the following inclusion criteria: people self-identifying as women between the ages of 18- and 50-years physically present at one of the two pharmacy locations, or at the outreach event. The screening form was developed using CDC criteria and included close-ended questions regarding the use of teratogenic medications, folic acid supplementation, alcohol, tobacco, recreational drugs, vaccinations received, past medical history, obstetric history, awareness of preconception health services being offered, and interest in receiving the services. It also included an item on the use of contraception. The form took 3–5 min to fill out.

Participants then received a personalized report that identified if they had intervention opportunities in any of the following categories: planning with birth control, tobacco and alcohol cessation, up to date vaccinations, folic acid supplementation, appropriate management of medical conditions, provision of prescription medication use, family and obstetric history, illicit drug use and weight management. Each category on the personalized report was classified as either “meeting goal” or “not meeting goal”, based on the presence of one or more factors as identified from responses on the screening form. This was followed by a subsequent recommendation for intervention based on clinical practice guidelines outlined in the CDC report on Improving Preconception Health and Health Care [35]. 

Data were manually extracted from the screening form and entered into a Microsoft Excel spreadsheet. These data included: age, medication history, vaccination history, disease history, lifestyle and behaviors, pregnancy history, patient’s readiness for pregnancy and their awareness of and interest in receiving preconception health services. Outcomes measured included the number of screened patients in need of preconception health services as defined by the presence of one or more of the following: lack of contraceptive use, any alcohol use, medical history indicating a high-risk condition (e.g., diabetes), body mass index >25 kg/m^2^, need for immunization(s), teratogenic medication use, and nicotine use. Need for immunization(s) was determined by anyone who did not indicate receiving any of the immunizations mentioned on the screening form (human papilloma virus (HPV); measles, mumps, rubella (MMR); varicella or chickenpox disease; influenza; tetanus, diphtheria, pertussis (Tdap)). Teratogenic medications were defined as agents known to cause birth defects and examples were listed on the screening form for patients to indicate if they were taking. Additional outcomes included the number of screened patients who were aware the pharmacy offered preconception health services, and if participants’ interest in receiving services differed between pharmacy locations and outreach event groups. The University of California San Diego Human Research Protections Program institutional review board reviewed the study protocol and certified this study as not human subject research.

Data analysis: Descriptive statistical tests in Excel were used to summarize the data and STATA 16.0 was used to analyze categorical data using Pearson’s chi-squared test. A *p*-value of less than or equal to 0.05 was considered significant. 

## 3. Results

A total of 43 women participated in the preconception health screening. The average age was 30 years, with ages ranging from 19 to 47 years. Patient characteristics are summarized in Table 1.

**Figure 1:**
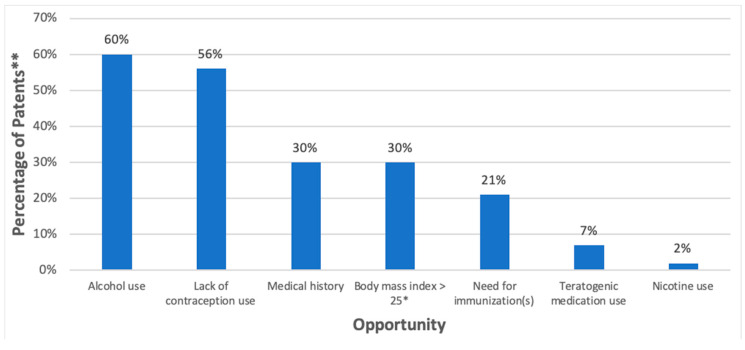
Opportunities for preconception health (*n* = 43). * kg/m^2^, ** Each individual could have more than one risk factor; does not total 100%.

Forty-two (98%) participants were identified as having at least one opportunity for preconception health services (Figure 1). Twenty-six participants (60%) reported alcohol use and 24 (56%) indicated they were not using contraception. Thirteen participants (30%) reported a past medical history of at least one chronic disease, including diabetes (*n* = 2, 5%), thyroid disease (*n* = 3, 7%), and anxiety and/or depression (*n* = 4, 9%). Other risk factors identified included a body mass index >25 kg/m^2^ (*n* = 13, 30%), use of the potentially teratogenic medications lorazepam (*n* = 2, 5%), clonazepam (*n* = 1, 2%), and topiramate (*n* = 1, 2%), and nicotine use (*n* = 1, 2%). Nine participants (21%) did not report receiving any of the immunizations on the screening form.

Eleven participants (26%) were aware that the pharmacy offers preconception health services and 24 (56%) were interested in learning more about the services offered. There was no significant difference between participants’ interest in receiving services as it relates to setting (16.7% in community pharmacies vs. 24.0% in outreach event, *p* = 0.34). Additional responses to the screening questionnaire items are shown in Table 2. 

## 4. Discussion

This study identified numerous opportunities in the community pharmacy setting for pharmacists to provide preconception care services. These opportunities included interventions involving alcohol use, contraception use, nicotine use, teratogenic medication use, weight management, immunizations, and management of high-risk medical conditions. Patient care services in these areas are important in ensuring safe environments for pregnancy, as well as overall health. This is especially true, as people with chronic medical conditions are at an increased risk for pregnancy-related complications, but most have little knowledge of the specific complications they are at risk for, even for those who previously experienced pregnancy complications [36]. Hence, pharmacists can step in to provide the necessary education. Furthermore, these interventions, such as education about alcohol use during the preconception period, may actually alter patients’ behavior. For example, Lassi and colleagues found that women who participated in preconception counseling significantly reduced their alcohol use during the first trimester compared to those who did not receive counseling (adjusted OR, 1.79; 95% CI, 1.08–2.97) [37]. Contrasting Reidenbach’s study, where the primary interventions identified were teratogenic prescription medication management and folic acid supplementation [34], the most prominent characteristics necessitating preconception care services in our sample were lack of contraception and alcohol use. One reason for this difference may be due to methodology—in Reidenbach’s study, participant data regarding teratogenic medications were also checked against their medication profile at the pharmacy. In our study, only participant responses were relied upon to indicate if they were taking potentially teratogenic medications.

In this study, many of the opportunities identified for preconception care fall within the scope of practice of a California community pharmacist. For example, since 2016, pharmacists in California have been able to prescribe self-administered hormonal contraceptives to patients without a prior prescription [38]. Pharmacists can also independently initiate and administer immunizations [39] and prescribe nicotine replacement products and devices for smoking cessation [40]. Although not directly tied to state legislation, other opportunities for preconception care identified in our study may also be addressed by pharmacists, including recommending alternative therapy for those on teratogenic medications, providing education and/or referrals for weight management and alcohol use, and ensuring patients have resources to manage chronic disease states such as diabetes and hypertension. Some pharmacists may work under collaborative practice agreements (CPAs) with other providers to perform additional disease state management activities [41]. 

Despite the evident need for preconception care, most participants were not interested in scheduling an appointment. This could be due to a number of different factors, including lack of prior exposure to this health information. De Jong-Potjer and colleagues found that a majority of women who received preconception counseling offers from their primary care providers, in the form of mailed letters, indicated they were interested [12]. In our study, there was only one point of contact, and there was no information provided to participants in advance. Furthermore, a general lack of understanding regarding preconception health could also contribute, as people may not see an immediate need for preconception care when they are not planning for pregnancy. Although the majority of participants did not indicate a reason for being uninterested in this service, a small number of participants (*n* = 5) did indicate they were not planning on becoming pregnant and therefore not interested in learning more about the preconception care services offered. Providing education on what preconception care involves may help. For example, a preconception care educational program implemented for women in a college setting found participants had increased knowledge of preconception health as indicated on posttest scores [42]. Finally, patients may lack a general awareness of any services provided in community pharmacies outside of medication dispensing. In a recent survey by Parker and colleagues, 89% of consumers surveyed (*n* = 9202) were aware pharmacies offered prescription ordering by phone or internet, but only 8% were aware that pharmacies also offer health screenings [43]. These factors may help explain why nearly 80% of our participants were not interested in making an appointment for preconception care services at the community pharmacy. With more consistent exposure and outreach from pharmacists regarding preconception health, and direct patient care services in general, people may be more likely to seek care in the community pharmacy setting. 

In order to increase utilization of preconception care services in the community pharmacy setting, additional studies should assess effective patient awareness strategies, continued expansion of pharmacists’ scope of practice, optimal patient education methods, and practical service delivery models. Further, addressing documented barriers to health behavior change should also be considered. These barriers include anxiety, stress, and challenges obtaining reputable information [44]. Finally, payment for pharmacist-provided patient care services remains a hurdle across the profession. Some avenues have emerged in certain states. For example, California authorized the state’s Medicaid provider, Medi-Cal, to cover selected pharmacist-provided services authorized by statewide protocols, including contraceptive care [45]. Payment for pharmacist contraception care is also available from Medicaid in Oregon and Maryland, and from some commercial plans in Oregon and Washington [46]. However, health insurance coverage of preconception care, regardless of which provider delivers services, is still a concern. According to the Association of Maternal and Child Health Programs (AMCHP) briefing on preconception health reform, few health plans provide any coverage specifically for preconception care services, and reimbursement is not consistent across states or plans [47]. Ultimately, without health insurance coverage, patients’ only option would be to pay out-of-pocket for preconception care services provided in the community pharmacy. This could impact their willingness and/or ability to receive these important services.

Limitations of this study include small sample size and sampling bias given respondents were selected based on their attendance to one of the aforementioned locations. In addition, not all those present at the time of data collection participated in the screening, and the number of participants that refused to complete the screening questionnaire was not collected, introducing a component of response bias.

## 5. Conclusions

Administering preconception health screenings in the community pharmacy setting provides opportunities to identify patients who may benefit from preconception care services. Patient awareness and interest in these services remains a barrier to providing them in a community pharmacy. Additional studies are needed to identify optimal strategies to enhance patient awareness and acceptance of pharmacist-provided preconception care services. 

## Figures and Tables

**Table 1:** Participant demographics (*n* = 43).

Characteristic	*n* (%)
Screening location	
Outreach event	25 (58%)
Pharmacy #1	10 (23%)
Pharmacy #2	8 (19%)
Age (years)	
18–19	1 (2%)
20–29	23 (54%)
30–39	13 (30%)
40–49	6 (14%)
Has health insurance	
Yes	33 (77%)
No	3 (7%)
No response	7 (16%)

**Table 2:** Participant responses to screening questionnaire (*n* = 43).

Item and Response	*n* (%)
Aware pharmacy offers preconception services	
Yes	11 (26%)
No	32 (74%)
Interested in learning more	
Yes	24 (56%)
No	19 (44%)
Why are you interested in these services *	
Planning to become pregnant in future	11 (26%)
Educational purpose	10 (23%)
Family members or friends planning to become pregnant	3 (7%)
Other	2 (5%)
Not applicable/No response	18 (42%)
Would like to schedule an appointment with pharmacist	
Yes	8 (19%)
No	34 (79%)
Not now, maybe in the future	1 (2%)
Why not interested in these services *	
Not planning on getting pregnant	5 (12%)
Time constraint	3 (7%)
Cost of service	1 (2%)
Lack of education	1 (2%)
Currently pregnant	1 (2%)
Other	5 (12%)
Reason not provided	27 (63%)

* Each individual could select more than one response.

## Supplementary Materials

The following are available online at https://www.mdpi.com/2226-4787/8/4/181/s1, File: Young Women’s Health Screening Form.
