# Supplementary material for: Patient Screenings for Preconception Health Interventions at a Community Pharmacy
Source: Pharmacy (Basel). 2020 Oct 5;8(4):181. doi: 10.3390/pharmacy8040181 (PMC7712468; doi:10.3390/pharmacy8040181)
Supplement: Supplementary file 1 [file pharmacy-08-00181-s001.pdf]

Young Women's Health Screening Form (For Women Up to Age 50)

Section 1: Patient Information

Name ____ Today's Date _________________

Age ________________ Height ____________________ Weight _____________________

Email _________________________________ Phone Number _________________________

BMI _________ Glucose Reading __________

Pharmacy ____________________________ Health Insurance ________________________

Section 2: Medication History

Please check the following medications/supplements you are currently on:

- ☐ Isotretinoin
- ☐ Folic Acid
- ☐ Levothyroxine
- ☐ Warfarin
- ☐ Xarelto
- ☐ Thalidomide
- ☐ Other anti-seizure medications ___________
- ☐ Other blood thinning medications ____________
- ☐ Diazepam
- ☐ Gabapentin
- ☐ Carbamazepine
- ☐ Levetiracetam
- ☐ Lorazepam
- ☐ Topiramate
- ☐ Valproic Acid
- ☐ Felbamate
- ☐ Phenobarbital
- ☐ Phenytoin

Are you currently on birth control?

- ☐ No
- ☐ Yes, I use: ___________________

Section 3: Vaccination History

Please check all of the following vaccinations you've received and what year:

- ☐ Human Papilloma Virus (HPV) ______
- ☐ Measles, Mumps, Rubella (MMR) ______
- ☐ Varicella (Chickenpox) or chickenpox disease ______
- ☐ Influenza Virus (Flu Shot) ______
- ☐ Tetanus, Diphtheria, Pertussis (Tdap) ______

Section 4: Medical History

Please check all of the following medial conditions you have been diagnosed with:

- ☐ HIV/AIDS
- ☐ Diabetes
- ☐ Thyroid Disease
- ☐ Folic Acid Deficiency
- ☐ STI (Chlamydia, Gonorrhea, etc.)
- ☐ Hypertension
- ☐ Seizure Disorder
- ☐ Bipolar Disorder
- ☐ Hepatitis B
- ☐ Maternal Phenylketonuria
- ☐ Obesity
- ☐ Severe Depression
- ☐ Eating Disorder
- ☐ Arthritis
- ☐ Schizophrenia
- ☐ Other Chronic Disease: ______________

Young Women's Health Screening Form (For Women Up to Age 50)

Section 5: Lifestyle and Behaviors

On average, how often do you use nicotine containing products each week?

☐ None ☐ 1-2 ☐ 3-4 ☐ 5 or more

On average how many alcoholic beverages do you drink each week?

☐ None ☐ 1-2 ☐ 3-4 ☐ 5 or more

Section 6: Pregnancy History

Please check all that apply:

☐ Miscarriage ☐ Infant Death
☐ Genetic Conditions ☐ Birth Defects
☐ Preterm labor (premature infant) ☐ Other ___________________________

Are you aware that your pharmacy offers preconception services?

☐ Yes ☐ No

Are you interested in learning more about preconception services offered at the pharmacy?

☐ Yes (Proceed to **A**) ☐ No (Proceed to **B**)

A. 1. Are you pregnant or trying to become pregnant in the next year?

☐ Yes ☐ No

2. Why are you interested in these services?

☐ Planning to become pregnant in the future
☐ Family members or friends planning to become pregnant
☐ Educational purpose
☐ Other ______________________________

3. Would you like to schedule an appointment with Dr. Sally Rafie?

☐ Yes ☐ No

B. 1. Why are you not interested in preconception care services?

☐ Cultural Beliefs ☐ Financial Status
☐ Time Constraint ☐ Other _______________________________
